# STAT1 is regulated by TRIM24 and promotes immunosuppression in head and neck squamous carcinoma cells, but enhances T cell antitumour immunity in the tumour microenvironment

**DOI:** 10.1038/s41416-022-01853-z

**Published:** 2022-05-20

**Authors:** Kelvin Anderson, Nathan Ryan, Divya Nedungadi, Felipe Lamenza, Michael Swingler, Arham Siddiqui, Abhay Satoskar, Puja Upadhaya, Maciej Pietrzak, Steve Oghumu

**Affiliations:** 1 Department of Pathology, The Ohio State University Wexner Medical Center, Columbus, OH USA; 2 Division of Anatomy, The Ohio State University Wexner Medical Center, Columbus, OH USA; 3 Department of Microbiology, The Ohio State University, Columbus, OH USA; 4 Department of Biomedical Informatics, The Ohio State University Wexner Medical Center, Columbus, OH USA

**Keywords:** Oral cancer, Tumour immunology

## Abstract

**Background:**

Head and neck squamous cell carcinoma (HNSCC) is a significant problem and is frequently resistant to current treatments. STAT1 is important in anti-tumour immune responses against HNSCC. However, the role of STAT1 expression by tumour cells and its regulation during HNSCC is unclear.

**Methods:**

We determined the effects of *STAT1* inhibition on tumour development and immunity in CAL27 and UMSCC22A HNSCC cell lines in vitro and in a HNSCC carcinogen-induced model in vivo.

**Results:**

*STAT1* siRNA knockdown in human HNSCC cells impaired their proliferation and expression of the immunosuppressive marker PD-L1. *Stat1*-deficient mice displayed increased oral lesion incidence and multiplicity during tumour carcinogenesis in vivo. Immunosuppressive markers PD-1 in CD8+ T cells and PD-L1 in monocytic MDSCs and macrophages were reduced in oral tumours and draining lymph nodes of tumour-bearing *Stat1*-deficient mice. However, STAT1 was required for anti-tumour functions of T cells during HNSCC in vivo. Finally, we identified TRIM24 to be a negative regulator of STAT1 that plays a similar tumorigenic function to STAT1 in vitro and thus may be a potential target when treating HNSCC.

**Conclusion:**

Our findings indicate that STAT1 activity plays an important role in tumorigenicity and immunosuppression during HNSCC development.

## Background

Head and neck squamous cell carcinoma (HNSCC) is the sixth most common form of cancer, affecting nearly one million people per year [1, 2]. With the annual burden of HNSCC expected to increase, there exists a dire need to identify and elucidate the context-dependent function of molecular targets that can be therapeutically exploited in cancer cells and associated cells of the HNSCC tumour microenvironment [3]. Signal transducer and activator of transcription 1 (STAT1) mediates the signalling of type I, II and III interferons (IFNs) and growth factors like epidermal growth factor and is involved in several biological processes, including apoptosis [4], inflammation [5] and cell cycle modulation [6, 7]. Not surprisingly, due to the wide range of biological activity mediated by STAT1, its activity is heavily altered in many HNSCC cases [8]. Interestingly, STAT1 downregulation has been implicated in oral carcinogenesis and immune escape, while STAT1 phosphorylation has been associated with increased survival [9–11].

Recent studies conducted by our group identified the critical anti-tumour role of STAT1 in the host immune response to HNSCC [12]. We used an orthotopic model of metastatic HNSCC cells injected into *Stat1*-deficient (*Stat1*^*−/−*^) or *Stat1*-sufficient (*Stat1*^*+/+*^) mice to show that mice deficient in *Stat1* are highly susceptible to tumour growth and metastasis. This was associated with an exhausted T cell phenotype with increased programmed cell death protein 1 (PD-1) expression, and increased accumulation of immunosuppressive myeloid-derived suppressor cell (MDSC) populations in the tumours of *Stat1*-deficient mice. These results indicate that host derived STAT1 is critical for anti-tumour immune responses against HNSCC. However, questions remain regarding the role of STAT1 expression by oral epithelial and cancer cells during stages of HNSCC tumour development.

Although STAT1 has long been considered to be a tumour suppressor by promoting HNSCC cell death [10], recent reports show that STAT1 activation also mediates programmed cell death ligand 1 (PD-L1) expression in HNSCC cells thereby promoting tumour immunosuppression [13, 14]. STAT1 activation has also been shown to mediate resistance to cisplatin therapy [15] and radioresistance in HNSCC cells [16]. A limitation to these studies that have investigated the impact of STAT1 activation in HNSCC cells are that the methods mostly used are in vitro cell culture models, xenograft models into nude mice or correlative studies from HNSCC clinical samples. These approaches generally do not address (i) the influence of STAT1 on the tumour microenvironment, (ii) the interaction of STAT1 expressing HNSCC cells with immune cells, stromal cells, and fibroblasts and (iii) the impact of STAT1 activation by oral epithelial cells on HNSCC tumour initiation and promotion. This represents a major gap in our knowledge of STAT1 activation by HNSCC cells in the tumour microenvironment, which is essential to advance our mechanistic understanding of the role of STAT1 in HNSCC, and design therapies that exploit the STAT1 pathway in HNSCC treatment.

Identifying cell-specific regulators of STAT1 activation in HNSCC cells can facilitate the development of novel approaches to HNSCC treatment. In this study, we identify a novel potential negative regulator of STAT1 in HNSCC cells—tripartite motif (Trim) 24 protein, also known as transcriptional intermediary factor 1. TRIM24 is a multifunctional protein that binds and regulates nuclear receptors, such as retinoic acid and oestrogen receptors, and associates with chromatin- and heterochromatin-associated factors that result in inactivation of *Stat1* transcription [17]. Thus, TRIM24 presents itself as a potential target for modulating STAT1 expression in the tumour microenvironment. Although TRIM24 has been shown to promote colorectal, ovarian and other cancers, [18–21], the effect of the TRIM24–STAT1 axis on HNSCC development still remains unclear.

To enhance our understanding of STAT1 activation and regulation in HNSCC initiation, growth and anti-tumour immune responses, we determined the effects of *STAT1* small interfering RNA (siRNA) knockdown on the human papillomavirus (HPV)-negative oral cancer cell lines CAL27 and UM-SCC22A. We also used the 4-nitroquinoline-1-oxide (4NQO) carcinogen-induced HNSCC model in STAT1-competent and STAT1-deficient mice to determine the effect of STAT1 on tumour development and immune responses to HNSCC in vivo [22]. This 4NQO mouse model recapitulates the mutational profile and immune landscape of non-HPV-associated human HNSCC and mirrors its development from early dysplastic to invasive stages [23]. Specifically, high incidence mutations observed in human HNSCC, such as in *Trp53*, *Notch1*, *Fat1*, *Lama3* and *Syne2*, were demonstrated to be associated with 4NQO-induced mouse tumours, while immune infiltration to tumours was maintained [23]. Indeed, STAT1 and IFN-γ activity are upregulated during PD-1 blockade in mice administered 4NQO [24]. Because of the similar mutational landscape of 4NQO-induced mouse oral carcinogenesis to human HNSCC, combined with the use of immunocompetent hosts, this model further allows for study of the immunologic determinants of HNSCC development [25]. Finally, we investigate the impact of *TRIM24* siRNA knockdown on STAT1 expression and subsequent cell proliferation, apoptosis and expression of immunosuppressive biomarkers in CAL27 and UM-SCC22A HNSCC cells. The findings of our study shed light on the role of STAT1 expression and regulation by HNSCC cells on tumour development and the immune response during HNSCC.

## Materials and methods

### Mice

*Stat1*^*+/+*^ and *Stat1*^*−/−*^ male and female BALB/c mice, 6–8 weeks old (*N* = 20, 10 males, 10 females per group) were used for experimental HNSCC studies. This sample size was selected to achieve 80% power to detect a 2.5-fold difference between groups (corresponding to an effect size of 2). Animals were kept according to regulations maintained by the University Laboratory Animal Resources. Experiments were approved by the Institutional Animal Care and Use Committee (Protocol #2018A00000054) and Institutional Biosafety Committee of The Ohio State University.

### Cell lines

Authenticated human HNSCC cell lines CAL27 (ATCC Cat# CRL-2095, RRID:CVCL_1107) and UM-SCC22A (Millipore Cat# SCC076, RRID:CVCL_7731) were used for these studies.

### siRNA knockdown

A reverse transfection mRNA knockdown cocktail containing STAT1 siRNA (Cell Signaling; Danvers, MA), TRIM24 siRNA (Life Technologies; Carlsbad, CA) or scrambled siRNA (Thermo Fisher; Waltham, MA) and 1 μL of Lipofectamine RNAiMAX in 100 μL OptiMEM serum-free media (Life Technologies; Carlsbad, CA) was prepared and plated at 100 μL per well. Following 15 min of room temperature incubation, cells were plated at 2.5 × 10^5^ cells/well in 500 μL antibiotic-free complete media in each well. Cells were allowed to grow for 48 or 72 h before downstream analyses.

### Western blot

In all, 30 μg protein was loaded into a 10% acrylamide SDS-PAGE gel (National Diagnostics; Atlanta, GA). Standard Western Blotting techniques were used as described previously [26]. Antibodies used included STAT1(14994T), phospho STAT1 (9167S), TRIM24 (79030S), MCM3 (4012S), cleaved CASP8 (8592T), GAPDH (2118S) (Cell Signaling), BCL2 (IMG-80093, IMGENEX) and KI67 (M7240, Agilent). Blots were incubated with goat anti-rabbit HRP-linked (31460, Thermo Fisher Scientific, Rockford, IL) or anti-mouse IgG HRP-linked (7076 S, Cell Signaling Technology) secondary antibodies and treated with Pierce ECL Western Blotting Substrate (Thermo Fisher; Waltham, MA).

### Enzyme-linked immunosorbent assay (ELISA)

IFN-γ ELISA was performed as described previously [27], using capture antibody (Biolegend, 505702, San Diego, CA) and detection antibody (505804).

### Carcinogen-induced HNSCC model

*Stat1*^*+/+*^ and *Stat1*^*−/−*^ (*n* = 20; 6–8 weeks old) BALB/c mice were administered the oral carcinogen 4NQO (Sigma Aldrich; St. Louis, MO) at a concentration 100 μg/mL in their drinking water for 16 weeks, followed by drinking water for 8 weeks [28, 29]. Control mice of each genotype were administered drinking water for the entire duration of the experiment. At 24 weeks following initial 4NQO treatment, mice were sacrificed. Tongue lesions and tumours were counted macroscopically and microscopically.

### Immunohistochemistry

Formalin-fixed-paraffin-embedded tissue sections were cut at a thickness of 5 µm and rehydrated. Heat-induced epitope retrieval was performed in sodium citrate antigen retrieval buffer. Tissues were stained with H&E or, for immunohistochemistry, Granzyme B (Abcam, ab4059; Cambridge, MA), Ki-67 (Abcam, Ab15580), PD1 (Cell Signaling 9664S), cleaved caspase 3 (Cell Signaling, 84651S), PD-L1 (Invitrogen, 14-5982-82; Waltham, MA), CD4 (Invitrogen, 14-0042-82), CD8 (Invitrogen, 14-0808-82) and CXCL9 (BioXCell, BE0309). Following the addition of primary antibody, the tissues were incubated with biotinylated goat anti-rabbit (Vector Laboratories, BA-1000; Burlingame, CA), biotinylated goat anti-hamster (Invitrogen, 31750), biotinylated goat anti-rat (Vector Laboratories, BA-9401) IgG (H + L) secondary antibodies or, for immunofluorescent staining, Alexa Fluor 488-conjugated goat anti-rabbit (Invitrogen, A11034) and Alexa Fluor 555 goat anti-rat (Invitrogen, A21434) with a DAPI counterstain. Regions of positive staining were quantified using ImageJ [26].

### Flow cytometry

Cells were stained with extracellular antibodies prior to fixation in 4% neutral-buffered formalin and permeabilisation. Fluorochrome-conjugated antibodies against CD4, CD8, CD11b, Ly6C, Ly6G, PD-1 and PD-L1 were used for extracellular staining. Intracellular staining was performed with antibodies against Gzmb, IFN-γ, pSTAT1 Y701 (Biolegend) and TRIM24 (AbClonal; Woburn MA). Mouse IgG1κ and Rat IgG1κ were used as isotype controls for Gzmb and IFN-γ, respectively. TRIM24 primary antibodies were further incubated with goat anti-rabbit FITC-conjugated secondary antibodies (BD Biosciences; San Jose, CA) prior to acquisition. Other samples were treated with propidium iodide (PI) and FITC-conjugated Annexin V for 15 min for apoptosis analysis. Samples were analysed using a FACS Celesta flow cytometer (BD Biosciences; San Jose, CA). Analysis was performed using the FlowJo software (FlowJo; Ashland, OR).

### Reverse transcription quantitative polymerase chain reaction (RT-qPCR)

RNA from cell lines was isolated using acid/chloroform/guanidinium thiocyanate/phenol extraction. Tongue samples were homogenised using a Bead Mill (VWR; Radnor, PA) before RNA extraction with DirectZol RNA miniprep (Zymo Research; Irvine, CA). cDNA was prepared from extracted RNA using a High-Capacity cDNA Reverse Transcription Kit (Applied Biosystems). RT-qPCR was performed on a CFX384 Real-Time PCR system (BioRad; Hercules, CA) using SYBR Green Master Mix (Thermo Fisher; Waltham, MA). Sequences of primers for *Bactin*, *Bcl2*, *Casp8*, *Cxcl9*, *Gapdh*, *Gzmb*, *Ifng*, *Ki67*, *Mcm3*, *Pd1* and *Pdl1* were generated using PrimerBank (https://pga.mgh.harvard.edu/primerbank/).

### Statistics

Analysis was performed in a blinded fashion. Statistical analyses were performed using GraphPad Prism v9.0 (GraphPad Software; San Diego, CA). Analysis of variance or Student’s *t* test (two-sided) was used to determine statistically significant differences between groups.

## Results

### STAT1 promotes expression of PD-L1 and proliferative biomarkers in HNSCC cells

**Fig. 1:**
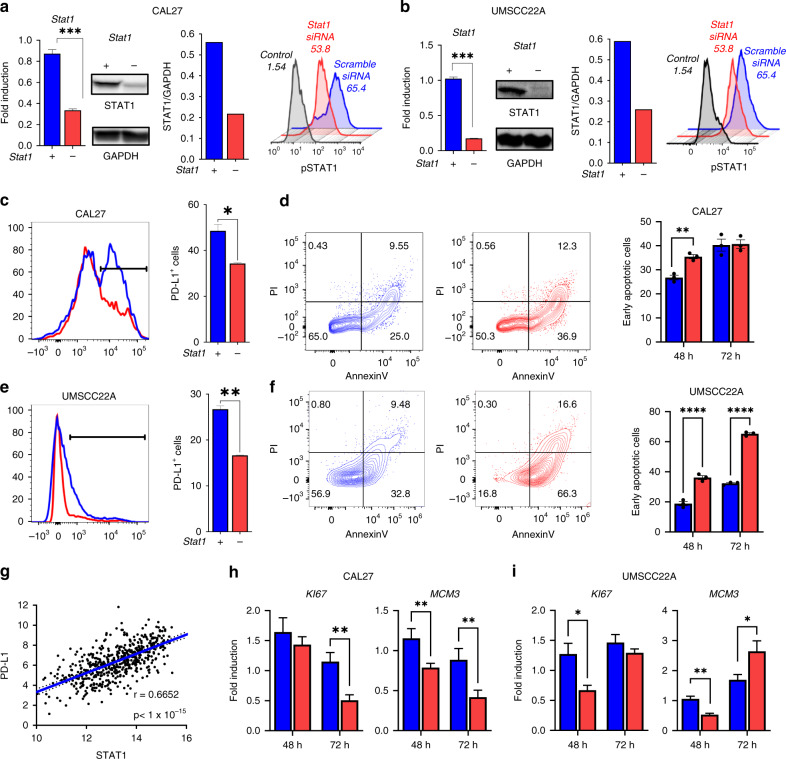
STAT1 promotes expression of PD-L1 and proliferative biomarkers in HNSCC cells. **a**, **b** Gene expression of *Stat1* determined by RT-qPCR, STAT1 and GAPDH protein levels, determined by western blot, and phospho STAT1 (pSTAT1) protein levels after IFNγ stimulation determined by flow cytometry, in *Stat1* siRNA and *scrambled RNA* treated **a** CAL27 and **b** UM-SCC22A cells. STAT1 and GAPDH protein levels were detected after 72 h of siRNA treatment, and western blot band intensity of STAT1:GAPDH were calculated in scramble and *Stat1* siRNA-treated cells. Numbers in histogram plots represent percentage of phospho STAT1 positive cells. Controls are unstimulated cells. **c**, **e** PD-L1 expression by **c** CAL27 and **e** UM-SCC22A cells after 72 h of siRNA treatment, determined by flow cytometry. **d**, **f** Flow gating and frequencies of AnnexinV^+^PI^−^ populations among **d** CAL27 and **f** UM-SCC22A cells after 72 h of siRNA treatment. **h**, **i** Gene expression of *Ki67* and *Mcm3*, determined by RT-qPCR in **h** CAL27 and **i** UM-SCC22A cells after 48 or 72 h siRNA treatment. Data are presented as mean +/− SEM. *p value <0.05; **p value <0.01; ***p value <0.001, ****p value <0.0001 between groups.

To explore the role of STAT1 in HNSCC, we performed siRNA mediated knockdown of *Stat1* expression in the human HNSCC cells. We used the CAL27 and UM-SCC22A cell lines because they are HPV negative HNSCC cells that express PD-L1 and STAT1. We first confirmed downregulation of *Stat1* mRNA transcripts and total STAT1 protein in both cell lines using RT-qPCR and western blot (Fig. 1a, b). Further, in CAL27 cells, *STAT1* siRNA knockdown decreased STAT1 phosphorylation compared to scrambled RNA treated cells. We did not observe any differences in STAT1 phosphorylation between *STAT1* siRNA and scrambled RNA treated UM-SCC22A cells (Fig. 1a, b**)**. Next, we determined the effects of STAT1 knockdown on immune suppression, apoptosis and biomarkers of cell proliferation. We first examined the effects of *Stat1* silencing on HNSCC cell PD-L1 expression by flow cytometry. We observed that PD-L1 surface expression was significantly downregulated following *Stat1* knockdown in both cell lines, indicating an essential role for STAT1 in promoting PD-L1 surface expression on HNSCC cells (Fig. 1c, e). Notably, despite the low constitutive expression of PD-L1 by UM-SCC22A cells, STAT1 knockdown eliminated the expression of this immunosuppressive receptor on this HNSCC cell line (Fig. 1e). Our data demonstrate that STAT1 promotes PD-L1 expression on HPV negative HNSCC cells in vitro. We further characterised the relationship between STAT1 and PD-L1 expression using the publicly available cancer genome atlas (TCGA) database of HNSCC patients. We observed a striking positive correlation between STAT1 and PDL1 in tumours of HNSCC patients (Fig. 1g), further supporting our hypothesis of PD-L1 regulation by STAT1 in HNSCC.

To examine the effects of STAT1 on apoptosis, we utilised Annexin-V/PI staining on cell populations 48 and 72 h following STAT1 siRNA knockdown. We observed a significantly higher percentage of early apoptotic Annexin-V^+^I^−^ cells in STAT1-deficient compared to STAT1-sufficient CAL27 and UM-SCC22A cell lines (Fig. 1d, f). Finally, we examined the effects of STAT1 on biomarkers of cell proliferation (*Ki67* and *Mcm3)* by RT-qPCR [30]. We observed decreased expression of both markers following STAT1 knockdown in CAL27 **(**Fig. 1h). In UM-SCC22A, we observed a similar downregulation of these proliferation markers at 48 h following STAT1 inhibition (Fig. 1i). Taken together, these data indicate that STAT1 expression by HNSCC cells promotes PDL1 expression, increases proliferative potential, and inhibits apoptosis, thereby enhancing the tumorigenicity of HNSCC cells.

### Global STAT1 deficiency in a 4NQO model of HNSCC results in increased incidence of carcinogenesis

**Fig. 2:**
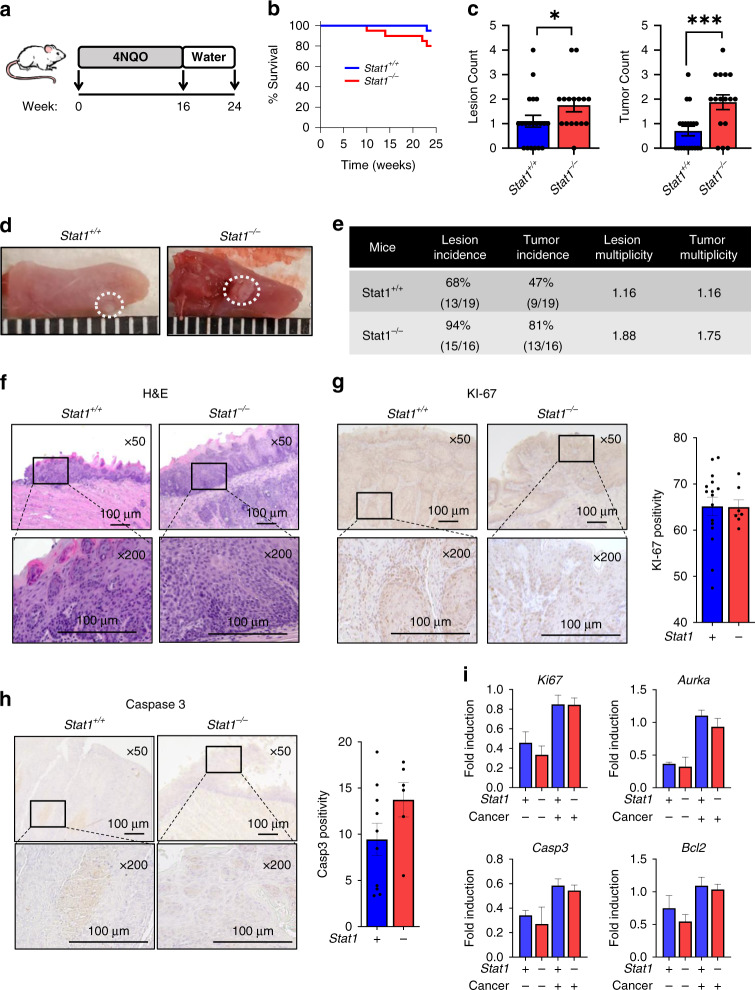
Global STAT1 deficiency in a 4NQO model of HNSCC results in increased incidence of carcinogenesis. **a** 4NQO model of carcinogen induced oral carcinogenesis showing time period of 4NQO administration and terminal sacrifice. **b** Kaplan–Meier curve depicting survival of 4NQO-treated *Stat1*^*+/+*^ and *Stat1*^*−/−*^ mice through the duration of the experiment. **c** Counts of lesions and tumours on the tongues of *Stat1*^*+/+*^ and *Stat1*^*−/−*^ 4NQO-treated mice determined at terminal sacrifice. **d** Representative images of mouse tongues containing tumours from 4NQO-treated *Stat1*^*+/+*^ and *Stat1*^*−/−*^ at terminal sacrifice. **e** Tabular illustration of the incidence and multiplicity of lesions and tumours in experimental mice. **f** Representative H&E-stained images of dysplastic lesions in tongues from *Stat1*^*+/+*^ and *Stat1*^*−/−*^ mice. **g**, **h** Representative immunohistochemistry images of oral lesions in tongues of *Stat1*^*+/+*^ and *Stat1*^*−/−*^ mice stained with **g** KI-67 and **h** cleaved caspase 3. Bar graphs depict regions of positive staining from 10 representative fields each for *n* = 6 or 8 mice per group. **i** Fold induction of *Ki67*, *Aurka*, *Casp3* and *Bcl2* transcripts in the tongues of control and carcinogen-induced *Stat1*^*+/+*^ and *Stat1*^*−/−*^ mice determined by RT-qPCR (*n* = 10 cancer-induced mice per group). Data are presented as mean +/− SEM. *p value <0.05; ***p value <0.005 between groups.

Previous work from our group demonstrated that in STAT1-sufficient murine HNSCC cells orthotopically injected into *Stat1*^+/+^ and *Stat1*^*−/−*^ mice, tumour growth is significantly enhanced when *Stat1* is deleted in host tissue [12]. Therefore, to determine the impact of tumour-derived STAT1 on HNSCC tumour development in vivo, we used the oral carcinogen 4NQO to induce oral carcinogenesis in both *Stat1*^+/+^ and *Stat1*^*−/−*^ mice (Fig. 2a). We chose this model because it recapitulates the initiation, promotion and progression stages of carcinogenesis in clinical cases of non-HPV associated HNSCC. First, we observed increased mortality in carcinogen induced *Stat1*^*−/−*^ compared to *Stat1*^*+/+*^ mice (Fig. 2b). At terminal sacrifice, we also noted higher incidence and multiplicity of both lesions and tumours in *Stat1*^−/−^ mice (Fig. 2c–e). Our findings were confirmed by H&E staining of the carcinogen-induced tongue tissue (Fig. 2f). To determine whether global STAT1 deficiency affects proliferation and apoptosis of tumour cells in vivo, we measured Ki-67 and caspase 3 expression in tumours by RT-qPCR and immunohistochemistry. We also measured Aurka and Bcl2 gene expression levels. Overall, proliferative and apoptotic markers were not affected by STAT1 deficiency in vivo (Fig. 2g–i). Taken together, our results indicate that despite the pro-tumorigenic properties of STAT1 in HNSCC tumour cells, global STAT1 deficiency results in higher tumour burdens in the carcinogen-induced model of HNSCC carcinogenesis. Although these results mimic our observations in the orthotopic model, the difference in tumour development between *Stat1*^+/+^ and *Stat1*^*−/−*^ mice in the 4NQO model was not as high as in the orthotopic model. These results demonstrate that, although STAT1 expression by immune cells is essential for an anti-tumour response, STAT1 expression by tumour cells might influence tumour development to a lesser extent than in immune cells, but in a manner that suggests a tumour promoting role in vivo.

### STAT1 promotes PD-1/PD-L1 expression in the tumour microenvironment during HNSCC

**Fig. 3:**
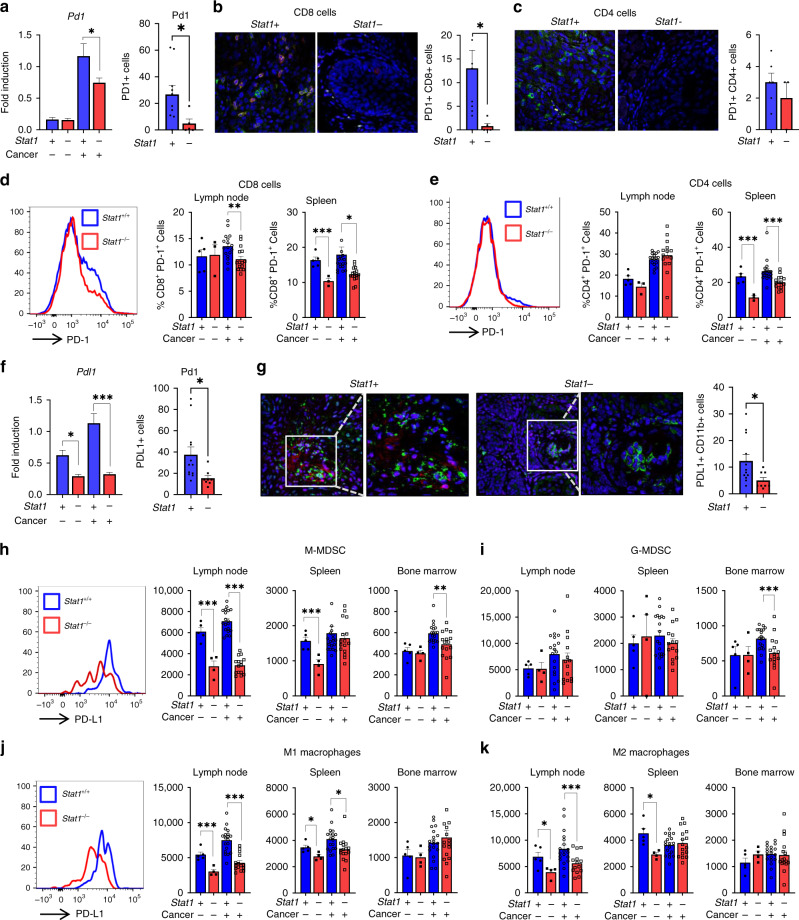
STAT1 promotes PD-1/PD-L1 expression in the tumour microenvironment during HNSCC. **a** Gene and protein expression of Pd1 in the tongues of experimental mice determined by RT-qPCR and immunofluorescence microscopy. **b**, **c** Representative immunofluorescence staining of **b** PD1 (green), CD8 (red) and **c** PD1 (green), CD4 (red) in tongue tumours of carcinogen-induced *Stat1*^*+/+*^ and *Stat1*^*−/−*^ mice. Bar graphs depict average numbers of PD1+CD8+ and PD1+CD4+ cells obtained from 10 representative fields each for *n* = 6 or 8 mice per group **d**, **e** PD-1 surface expression by **d** CD8+ cells and **e** CD4+ cells from the lymph nodes and spleens of experimental mice. Graph shows average percentage of PD1+CD8+ and PD1+CD4+ cells (*n* = 15 or 17 cancer induced mice per group). **f** Gene and protein expression of Pdl1 in the tongues of experimental mice determined by RT-qPCR and immunofluorescence microscopy. **g** Representative immunofluorescence staining of PDL1 (green) and CD11b (red) in tongue tumours of carcinogen-induced *Stat1*^*+/+*^ and *Stat1*^*−/−*^ mice. Bar graph depicts average numbers of PDL1+CD11b+ cells obtained from 10 representative fields each for *n* = 6 or 8 mice per group **h**, **i** Comparative histograms of PD-L1 surface expression by **h** M-MDSCs and **j** M12 macrophages in carcinogen-induced *Stat1*^*+/+*^ and *Stat1*^*−/−*^ mice. Graphs show mean fluorescence intensities (MFI) of PD-L1 surface expression by **h** M-MDSCs, **i** G-MDSCs, **j** M1 macrophages and **k** M2 macrophages in lymph nodes, spleens and bone marrow of experimental mice (*n* = 15 or 17 cancer-induced mice per group). Data are presented as mean +/− SEM. *p value <0.05; **p value <0.01; ***p value <0.001 between groups.

Given the observed role for STAT1 in PD-L1 expression by HNSCC cells in vitro, we next explored the impact of STAT1 on PD-1 and PD-L1 expression in the tumour microenvironment during HNSCC carcinogenesis in vivo. We observed significantly reduced gene expression and cell protein expression of Pd1 at the tongue tissues of carcinogen induced *Stat1*^*−/−*^ mice, indicating decreased PD-1 immune checkpoint activity (Fig. 3a). We had previously observed that PD-1 was strongly induced in T-lymphocytes of tumour-bearing *Stat1*^*−/−*^ mice orthotopically injected with *Stat1* competent aggressive HNSCC [12]. Therefore, we examined PD-1 expression in T lymphocytes of carcinogen-induced *Stat1*^*+/+*^ and *Stat1*^−/−^ mice. In contrast with *Stat1*^*−/−*^ orthotopic tumour-bearing mice, PD-1 expression was reduced in CD8^+^ T cells in the tumours, lymph nodes and spleens of carcinogen-induced *Stat1*^*−/−*^ mice compared to *Stat1*^*+/+*^ mice (Fig. 3b, d). PD-1 expression in CD4^+^T cells was reduced in spleens, but not tumours and draining lymph nodes of carcinogen-induced *Stat1*^*−/−*^ mice compared to *Stat1*^*+/+*^ mice (Fig. 3c, e). Combined with previous studies, these data suggest that during HNSCC, induction of PD-1 expression on T cells is partly dependent on STAT1 expression by tumour cells.

*Pdl1* gene expression was similarly reduced in tongue tissues of carcinogen induced *Stat1*^*−/−*^ mice (Fig. 3f). Because PD-L1 expression is induced in myeloid cells during HNSCC, we analysed the role of STAT1 in PD-L1 expression by CD11b^+^ myeloid populations including Ly6C^int^ Ly6G^+^ cells, Ly6C^hi^ Ly6G^−^ cells, classical M1 macrophages (Ly6C^−^F4/80^+^CD206^−^), and alternatively activated M2 macrophages (Ly6C^−^F4/80^+^CD206^+^) from the lymph nodes, spleens, and bone marrow of carcinogen induced *Stat1*^*−/−*^ and *Stat1*^*+/+*^ mice. PD-L1 expression was reduced among CD11b+ cells in the tumours of *Stat1*^*−/−*^ mice (Fig. 3g). Ly6C^hi^ Ly6G^−^ cells, M1, and M2 macrophages from the draining lymph nodes of *Stat1*^*−/−*^ mice demonstrated lower PD-L1 expression compared to *Stat1*^*+/+*^ mice (Fig. 3h–k). In the spleen, we observed a decrease in PD-L1 among M1 macrophages in *Stat1*^*−/−*^ mice, although we did not observe any differences among other immunosuppressive myeloid populations (Fig. 3j). Examination of myeloid cells in the bone marrow showed diminished PD-L1 expression by Ly6C^int^ Ly6G^+^ and Ly6C^hi^ Ly6G^−^ cells (Fig. 3h, i). Taken together, our findings suggest that tumour-derived STAT1 is required for PD-1/PD-L1 immune checkpoint expression and immune suppression during experimental HNSCC in vivo.

### STAT1 is required for anti-tumour function by T-lymphocytes during HNSCC carcinogenesis

**Fig. 4:**
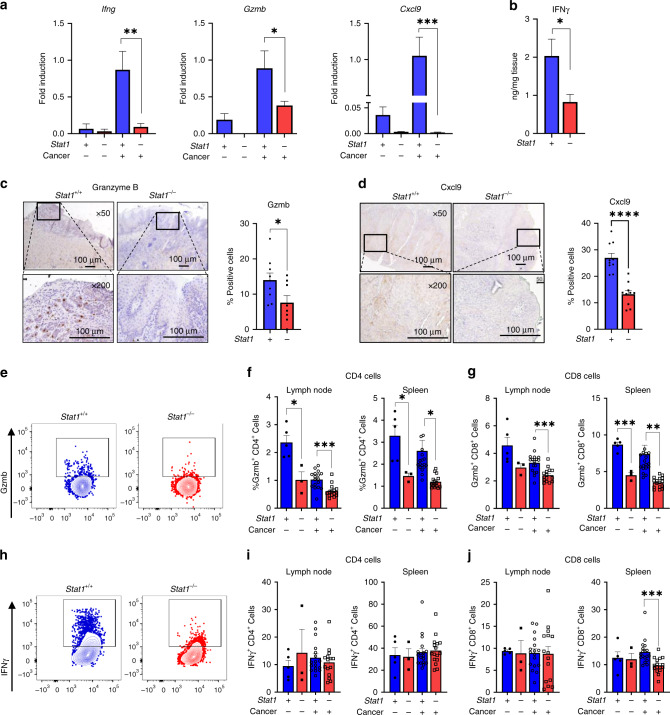
STAT1 is required for anti-tumour function by T-lymphocytes during HNSCC carcinogenesis. **a** Fold induction of *Ifng*, *Gzmb* and *Cxcl9* transcripts in the tongues of experimental mice determined by RT-qPCR. **b** IFNγ protein expression in tongue lysates of carcinogen-induced *Stat1*^*+/+*^ and *Stat1*^*−/−*^ mice as determined by ELISA. **c**, **d** Representative immunohistochemistry images of oral tumours in tongues of *Stat1*^*+/+*^ and *Stat1*^*−/−*^ mice stained with **c** Gzmb and **d** Cxcl9. Bar graphs depict regions of positive staining from 10 representative fields each for *n* = 6 or 8 mice per group. **e**–**j** Representative dot plots of **e** Gzmb and **h** IFNγ in CD4+ T cells in draining lymph nodes of carcinogen-induced *Stat1*^*+/+*^ and *Stat1*^*−/−*^ mice as determined by intracellular flow cytometry. Graphs depict average percentage of **f** Gzmb+ CD4+, (G) Gzmb+ CD4+, **i** IFN-γ+CD4+, and **j** IFN-γ+CD8+ T cells in draining lymph nodes and spleens of cancer induced and control *Stat1*^*+/+*^ and *Stat1*^*−/−*^ mice (*n* = 15 or 17 cancer-induced mice per group). Data are presented as mean +/− SEM. *p value <0.05; **p value <0.01; ***p value <0.001 between groups; ****p value <0.0001.

Despite the increased PD-1/PD-L1 expression observed in carcinogen induced *Stat1* competent mice, tumour development was still lower than in *Stat1*^*−/−*^ mice. Therefore, we next examined potential STAT1 mediated anti-tumour mechanisms. Specifically, we measured IFN-γ, GranzymeB, and CXCL9 [31, 32], markers known to be regulated by STAT1 and are associated with T cell anti-tumour activity, effector responses and chemotaxis of anti-tumour effector T cells. At the primary tumour site, we found that *Gzmb, Ifng*, and *Cxcl9* transcripts were significantly reduced in carcinogen-induced *Stat1*^*−/−*^ compared to *Stat1*^*+/+*^ mice (Fig. 4a). ELISA analysis of tongue lysates and immunohistochemical staining of tongue lesion sites further corroborated the decreased IFN-γ, GranzymeB and Cxcl9 protein expression in *Stat1*^*−/−*^ mice (Fig. 4b–d). Given the decreased anti-tumour activity in the STAT1-deficient tumour microenvironment, we analysed T-cell populations of the oral draining lymph nodes and spleens of experimental mice. At the lymph nodes, Gzmb expression by CD4^+^ and CD8^+^ lymphocytes were decreased in *Stat1*^*−/−*^ mice (Fig. 4e–g). IFN-γ production was decreased in splenic CD8^+^ T cells and NK cells of the lymph nodes and spleen of carcinogen-induced *Stat1*^*−/−*^ mice compared to carcinogen-induced *Stat1*^*+/+*^ mice (Fig. 4h–j and Supplementary Fig. 1). These results demonstrate the importance of STAT1 in maintaining an effective anti-tumour immune response and immunosurveillance during HNSCC, and explain potential mechanisms underlying the increased tumour burden observed in *Stat1*^*−/−*^ mice compared to their *Stat1*^*+/+*^ counterparts.

### TRIM24 is selectively expressed in HNSCC cells and negatively regulates STAT1

**Fig. 5:**
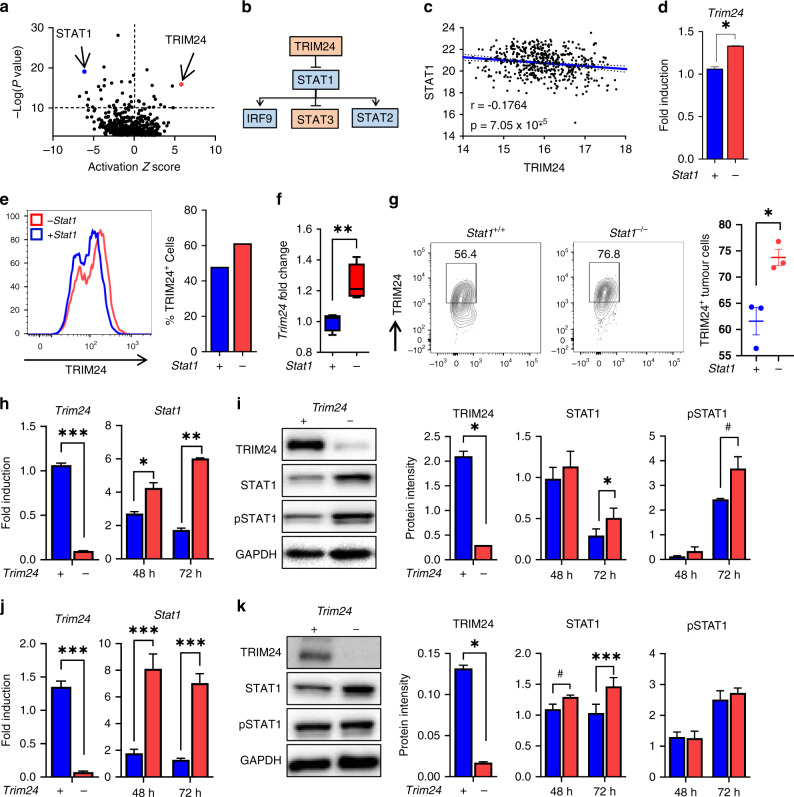
TRIM24 is selectively expressed in HNSCC cells and negatively regulates STAT1. **a** Volcano plot showing top 100 upstream regulators based on Ingenuity pathway analysis of RNA sequencing data from oral tumour bearing *Stat1*^*+/+*^ and *Stat1*^*−/−*^ mice (*n* = 4 per group). **b** Predicted mechanistic network analysis showing transcriptional targets of TRIM24 based on Ingenuity pathway analysis of RNA sequencing data from oral tumour-bearing *Stat1*^*+/+*^ and *Stat1*^*−/−*^ mice. Genes in red are upregulated and blue are downregulated in the RNA sequencing data set. **c** Correlation of TRIM24 expression levels with STAT1 expression in tumours from HNSCC patients samples acquired from The Cancer Genome Atlas. Significance determined by Pearson correlation coefficient. **d** Gene expression of *Trim24* determined by RT-qPCR in CAL27 cells treated with scramble or *Stat1* siRNA for 72 h. **e** TRIM24 protein expression by CAL27 cells treated with scramble or *Stat1* siRNA for 72 h, determined by flow cytometry. **f** Gene expression of TRIM24 in tumours of HNSCC tumour-bearing mice orthotopically injected with the LY2 cell line. **g** TRIM24 protein expression, determined by flow cytometry, in HNSCC cells of mice orthotopically injected with the LY2 cell line. **h**, **j** Gene expression of *Trim24* and *Stat1* determined by RT-qPCR in (H) CAL27 and (J) UMSCC22A cells treated with scramble or *Trim24* siRNA for 48 or 72 h. **i**, **k** Protein expression of TRIM24, STAT1, pSTAT1 and GAPDH, determined by western blot, in **i** CAL27 and **k** UMSCC22A cells accompanied by the protein band intensities relative to GAPDH in scramble and *Trim24* siRNA-treated cells after 48 and 72 h (*n* = 3 samples per group). Data are presented as mean +/− SEM. ^#^*p* value <0.1, *p value <0.05; **p value <0.01; ***p value <0.001 between groups.

We next investigated potential STAT1 modulators that could selectively impact STAT1 signalling in HNSCC cells. TRIM24 has previously been shown to be over expressed in advanced HNSCC and is associated with HNSCC metastasis [19]. Pathway analysis of RNA sequencing data from tumours from *Stat1*^*+/+*^ and *Stat1*^*−/−*^ mice orthotopically injected with LY2 HNSCC cells also identified TRIM24 as the top predicted negative transcriptional regulator of STAT1 during HNSCC in our data set (*z* score –5.804, *p* value 1.32 × 10^−16^) (Fig. 5a, b). Mutational analysis of these orthotopically injected tumours in *Stat1*^*+/+*^ and *Stat1*^*−/−*^ mice from sequencing data are shown in Supplementary Fig. 2. Analysis of 519 HNSCC patients from the cancer genome atlas (TCGA) did not show a real inverse correlation between TRIM24 and STAT1 expression (*r* = −0.1764, *p* = 7.05 × 10^−5^) (Fig. 5c). However, in *STAT1*-knockdown CAL27 HNSCC cells, gene and protein expression of *TRIM24* is slightly, but significantly increased (Fig. 5d, e). Interestingly, in our orthotopic HNSCC model, we found that TRIM24 expression is high in HNSCC cells but not in tumour-infiltrating T lymphocytes (Fig. 5f, g), suggesting the potential of targeting TRIM24 to modulate STAT1 signalling specifically in HNSCC cells but not T cells. Therefore, we investigated the effects of TRIM24 in human HNSCC cells using *TRIM24* specific siRNA knockdown. We first verified knockdown of TRIM24 gene and protein expression by RT-qPCR and Western blot (Fig. 5h, i). Next, we determined the effect of TRIM24 on STAT1 expression, and we observed a marked increase in *STAT1* transcripts (Fig. 5h), total STAT1 and phosphorylated STAT1 protein (Fig. 5i and Supplementary Fig. 3). These data strongly implicate TRIM24 as a negative regulator of STAT1 production and activity in HNSCC cells. These results were further corroborated in UMSCC22A cells, although p-STAT1 protein expression was unchanged in TRIM24 knockdown cells (Fig. 5j, k).

### Effects of TRIM24 on HNSCC cells are mediated by STAT1-dependent and independent mechanisms

**Fig. 6:**
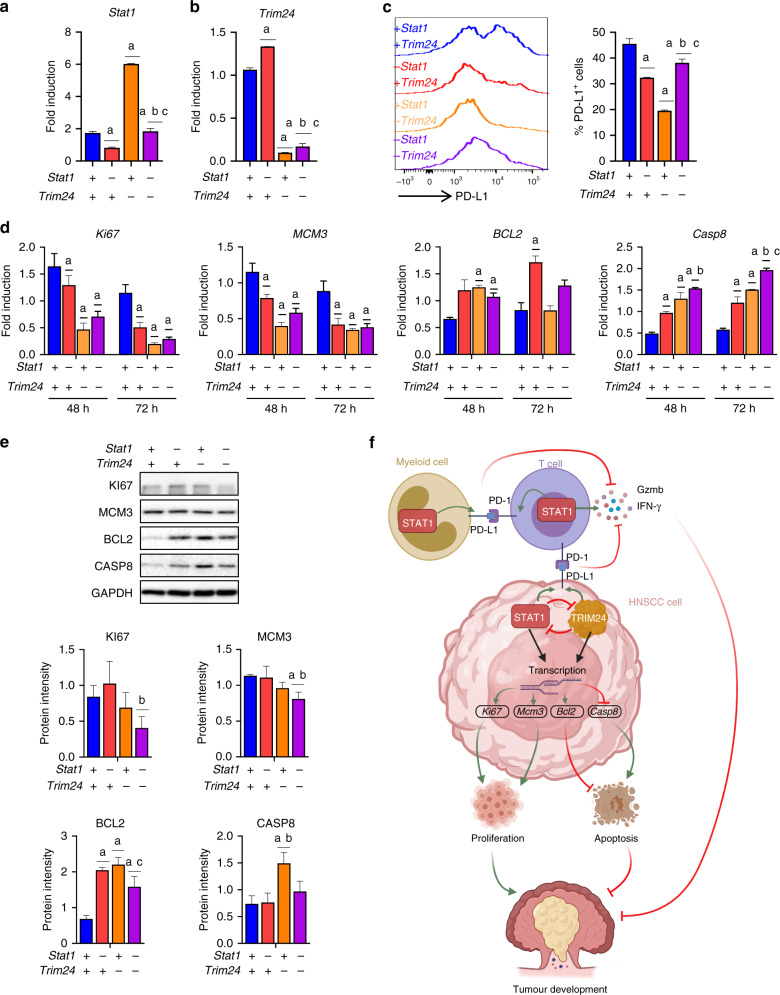
Effects of TRIM24 on HNSCC cells are mediated by STAT1-dependent and independent mechanisms. **a**, **b** Gene expression of **a** *Stat1* and **b** *Trim24* determined by RT-qPCR in CAL27 cells at 72 h post-siRNA treatment of cells treated with scramble, *Stat1*, *Trim24* and *Stat1/Trim24* siRNA. **c** PD-L1 protein expression by siRNA-treated CAL27 cells, determined by flow cytometry. **d** Gene and **e** protein expression of Ki67, Mcm3, Bcl2, and Casp8, determined by RT-qPCR and Western blot in siRNA-treated CAL27 cells at 48 or 72 h. **f** Graphical model demonstrating the influence of STAT1 and TRIM24 on gene/protein expression and cell function in myeloid cells, T cells and HNSCC cells. Statistical significance (*p* < 0.05) in comparison to a +*Stat1*+*Trim24*, b −*Stat1*+*Trim24* and c +*Stat1*−*Trim24* is noted above each group (*n* = 5 biological replicates per group for RT-PCR and 3 biological replicates per group for Western blot). Data are presented as mean +/− SEM.

Given the co-inhibitory effects of TRIM24 and STAT1 expression in HNSCC cells, (Fig. 6a, b), we further explored the interaction between TRIM24 and STAT1 on HNSCC cell PD-L1 expression, apoptosis and cell proliferation. Interestingly, despite the increase in STAT1 induced by *TRIM24* knockdown, we observed that *TRIM24* silencing inhibited the tumour promoting effects and enhanced the tumour inhibitory effects of STAT1 activation. Specifically, while we showed STAT1 to be important for PD-L1 expression, we surprisingly saw that *TRIM24* knockdown resulted in an even more significant decrease in PD-L1 surface expression in CAL27 cells compared to *STAT1* siRNA treated cells (Fig. 6c). Similarly, *TRIM24* knockdown significantly decreased the expression of the proliferation markers *KI67* and *MCM3*, which we had showed to be decreased by *STAT1* inhibition (Fig. 6d, e and Supplementary Fig. 3). This was also true of the increased expression of the apoptotic markers *CASP8* and *BCL2* in *TRIM24* knockdown CAL27 and UMSCC22A cells (Fig. 6d, e and Supplementary Fig. 3). These results indicate that TRIM24, while a negative regulator of STAT1 in HNSCC cells, inhibits the tumour-promoting effects and enhances the tumour-inhibitory effects of STAT1 activation.

Next, we performed double knockdown experiments of *STAT1* and *TRIM24* to determine mechanisms of TRIM24 effects on HNSCC cells and the involvement of STAT1 activity. We determined the expression of PDL1 on *TRIM24* and *STAT1* single or double knockdown CAL27 cells. We observed that knockdown of *STAT1* abrogated the effects of *TRIM24* on PDL1 expression in these cells. On the other hand, knockdown of *TRIM24* did not significantly affect PDL1 expression on *STAT1* knock down cells (Fig. 6c). This suggests that the effects of TRIM24 on PDL1 expression in CAL27 HNSCC cells are STAT1 dependent. We also examined the impact of *TRIM24* and *STAT1* double knockdown HNSCC cells on markers of apoptosis and cellular proliferation. We observed similar or enhanced reduction of the proliferation markers Ki67 and *MCM3* in double knockdown compared to single knock down CAL27 and UMSCC cells (Fig. 6d, e and Supplementary Fig. 3). Taken together, our results demonstrate a unique interaction between TRIM24 and STAT1 in HNSCC cells. Based on these findings, we propose a model demonstrating the divergent roles of STAT1 signalling in cancer cells and immune cells of the tumour microenvironment (Fig. 6f). We also demonstrate the interactions of TRIM24 as an upstream regulator of STAT1, inhibiting STAT1 expression and phosphorylation in HNSCC cells. Conversely, STAT1 inhibits TRIM24 expression, although to a lesser extent (Fig. 6f). Further we demonstrate that the effects of TRIM24 on immunosuppression, apoptosis and proliferation of HNSCC cell are mediated by STAT1-dependent and independent mechanisms.

## Discussion

This study highlights new mechanistic insights into the role of STAT1 expression by HNSCC cells and immune cells, as well as the function of a novel STAT1 negative regulator TRIM24, in HNSCC cells. Using RNAi silencing technology, we found that STAT1 plays an essential role in PD-L1 expression by HNSCC cells. We further found, using the 4NQO murine model of HNSCC, that STAT1 signalling is essential for PD-1/PD-L1 expression in the tumour microenvironment and by immune cells, including the accumulation of immunosuppressive populations during head and neck carcinogenesis. We further demonstrate an important role for STAT1 in anti-tumour immunity by T cells, which supports our previous reports [12]. Lastly, we show a negative co-regulatory function between STAT1 and TRIM24, which together induce PD-L1 expression in HNSCC cells (Fig. 6f).

The results of our study indicate a pro-tumour role for tumour-derived STAT1 expression in HNSCC based on its ability to enhance PD-L1-mediated immunosuppression and proliferation in both UM-SCC22A and CAL27 HNSCC cell lines. The role of STAT1 in regulating apoptosis, however, appears to be context-dependent. A downregulatory effect of STAT1 on the anti-apoptotic protein BCL2 has been previously established, although not in HNSCC cells [33]. We show that following STAT1 silencing in CAL27 cells, BCL2 expression is highly upregulated, showing that STAT1 maintains its downregulatory effect on BCL2 among the HNSCC cells tested. On the other hand, STAT1 activity appears to induce caspase-8 expression [34]. However, the non-canonical elevation of *Casp8* expression we observed in CAL27 cells following STAT1 inhibition warrants further investigation, as this may be a driver behind tumour survival in cases of HNSCC with high expression of STAT1.

The role of tumour-derived STAT1 in promoting of PD-L1 expression, however, is more evident. Previously, our group observed that STAT1-competent HNSCC induced greater expression of both PD-1 and PD-L1 in immune cells of *Stat1*^*−/−*^ mice compared to *Stat1*^*+/+*^ in both in situ and metastatic orthotopic models of HNSCC [12]. Our current results show that global STAT1 deficiency in a carcinogen-induced model of HNSCC leads to decreased PD-1/PD-L1 expression in the tumour microenvironment and by immune cells. In light of the finding that PD-1/PD-L1 expression STAT1-deficient immune cells were elevated in the context of STAT1-competent tumours but not STAT1-deficient tumours, it appears that STAT1 expression by tumour cells plays a direct role in inducing immune checkpoint signalling between immune cells. Moreover, host immune cells do not require STAT1 to express high levels of PD-1 or PD-L1. Taken together, these findings indicate that PD-1/PD-L1 activity in immune cells is dependent on STAT1 expression by tumour cells during HNSCC but may be independent of STAT1 expression by immune cells.

It was surprising to observe a reduced accumulation of immunosuppressive myeloid populations in carcinogen induced *Stat1*-deficient mice compared to *Stat1*-sufficient mice. This contrasts with our previous findings where these populations significantly accumulate in *Stat1*-deficient mice bearing *Stat1* competent aggressive HSNCC tumours [12]. While it has been shown that STAT1 promotes M2 macrophage activity and development [35, 36], we previously observed that these populations also accumulate in *Stat1*-deficient mice. Our observation that these populations are reduced in the bone marrow of carcinogen induced *Stat1*^*−/−*^ mice implies that tumour-derived STAT1 plays a major role. Notably, we observed a significant decrease in Ly6C^hi^ Ly6G− cells, but not Ly6C^int^ Ly6G^+^ cells, in the lymph nodes and spleens of carcinogen induced *Stat1*-deficient mice, which is another population we previously observed to accumulate in *Stat1*-deficient mice bearing Stat1-competent aggressive HNSCC. Together, these results highlight the important role of tumour-derived STAT1 in promoting the accumulation of immunosuppressive myeloid populations, especially when these host derived immune cells are *Stat1* deficient. Because M-MDSCs are known to facilitate the epithelial-to-mesenchymal transition that precedes metastasis [37], tumour-derived STAT1 may be a predictor of immune suppression and metastasis in HNSCC. Alternatively, MDSC populations have been shown to expand in response to chronic inflammation [38, 39], and their deficiency may be due to the failure of *Stat1*^*−/−*^ mice to initiate a robust anti-tumour response during the early stages of head and neck carcinogenesis [40].

Despite the reduction in the immunosuppressive PD-1/PD-L1 axis and MDSC populations observed in carcinogen induced *Stat1*-deficient mice, they still displayed significantly higher mortality and burden of dysplasia and neoplasia compared to *Stat1* competent mice. This finding may be explained by our findings of decreased expression of Gzmb and IFN-γ by lymphocytes in *Stat1*^*−/−*^ mice, which are known to facilitate the anti-tumour immune response to HNSCC and inhibit tumour proliferation [41–43]. Initially, this observation may seem to contradict the heightened PD-1/PD-L1 signalling which is known to inhibit lymphocyte anti-tumour functions [14]. However, the global reduction of Gzmb in *Stat1*^*−/−*^ mice can be explained by the role STAT1 plays in its production in CD8 T cells [44]. Alternatively, decreased PD-1 expression by T-cells may be resultant of decreased activation rather than a direct result of STAT1 deficiency. Further, absent IFN-γ signal transduction mediated by STAT1 leads to failure to prepare immune cells for sufficient anti-tumour immune activity, as evidenced by decreased IFN-γ expression in the tumour microenvironment. However, we also observed a corresponding decrease in PD-L1 in the tumour microenvironment, which corresponds to the role of IFN-γ and STAT1 signalling in the production of PD-L1. Thus, while immune cell STAT1 activity is strongly associated with HNSCC survival and can be considerably variable among patients [11], our data supports the hypothesis that STAT1 activation by HNSCC cells could simultaneously potentiate immunosuppressive and tumorigenic effects during HNSCC. These data taken together indicate that while impaired STAT1 signalling within lymphoid populations is likely to produce a deleterious effect in HNSCC, selective targeting of STAT1 signalling in HNSCC cells could potentially enhance anti-tumour immune responses and inhibit tumour cell growth.

TRIM24 is a transcriptional intermediary factor that we have found to negatively correlate with STAT1 expression. Thus TRIM24 presents itself as an interesting target for modulating STAT1 expression and downstream effects in HNSCC. Previously, TRIM24 has been shown to bind to retinoic acid responsive elements of the STAT1 promoter, thereby repressing STAT1 transcription [17, 19]. However, there has been a long-standing debate on whether TRIM24 functions as either a tumour suppressor or oncogene, and the answer appears to be context-dependent [18, 45]. Notably, TRIM24 expression has been shown correlate with both locally advanced and metastatic HNSCC, though the mechanisms underlying this effect remain unclear [19, 46]. Although we did not observe a strong correlation between STAT1 and TRIM24 based on TCGA data, our results demonstrate that TRIM24 negatively regulates STAT1, and we present novel evidence that STAT1 also negatively regulates TRIM24 in HNSCC cells.

Despite its inhibitory effect on STAT1 expression, we also found TRIM24 to have a significant impact on the immunosuppressive ability of HNSCC cells, indicating a complex regulatory interaction beyond simple co-inhibitory effects. For example, while STAT1 is involved in PD-L1 expression, TRIM24 knockdown in HNSCC cells significantly inhibited PD-L1 expression to an even greater extent than observed in STAT1 knockdown HNSCC cells. This finding was particularly surprising given the observed STAT1 mediated promotion of PD-L1 expression and the downregulatory effect of TRIM24 on STAT1 protein expression and phosphorylation. These results appear to highlight the complex crosstalk between TRIM24 and STAT1 to induce PD-L1 expression in HNSCC. This is further demonstrated by our observation that STAT1 depletion in TRIM24 knockdown cells partially restored PD-L1 expression levels, indicating that TRIM24 effects of PD-L1 expression on HNSCC cells are at least partly mediated by STAT1. Potential transcriptional, translational and post-translational mechanisms underlying the complex interplay between TRIM24 and STAT1 in mediating immunosuppression in HNSCC cells will require further investigation.

Given that TRIM24 was selectively expressed in tumour cells of the HNSCC microenvironment potentially provides an opportunity for selectively targeting STAT1 and mitigating its tumour promoting effects in HNSCC in vivo. This is particularly significant since STAT1 mediates tumour promoting and inhibitory roles in HNSCC cells despite playing a significant anti-tumour function in immune cells. Further, the tumour inhibitory effects mediated by TRIM24 inhibition were significantly greater than STAT1 inhibition. There is therefore a strong rationale to exploring TRIM24 as a target in HNSCC treatment, which can potentially leverage the STAT1 pathway to improve HNSCC tumour outcomes. It is possible that TRIM24 inhibition could directly or indirectly inhibit the tumorigenicity of HNSCC tumour cells. Additionally, while TRIM24 expression is low in tumour-infiltrating lymphocytes, its downregulation could, in turn, upregulate STAT1 anti-tumour activity. A small molecule inhibitor of TRIM24 has been reported with high potency and favourable pharmacokinetic properties—a therapeutic role in HNSCC could be further explored in future studies [47].

In conclusion, our findings indicate that tumour-derived STAT1 regulates HNSCC tumorigenicity and PD-L1 immune checkpoint signalling, while T-cell-derived STAT1 is necessary for immune activation and anti-tumour immune responses. We further demonstrate that TRIM24 potentiates the tumour promoting function of STAT1 in HNSCC cells.

## Supplementary information

Supplemental Figures

Reproducibility Checklist

## Data Availability

All raw data supporting the findings of this study are available upon request. Sequencing data are available via the Gene Expression Omnibus (GSE198790).
