# Supplementary material for: STAT1 is regulated by TRIM24 and promotes immunosuppression in head and neck squamous carcinoma cells, but enhances T cell antitumour immunity in the tumour microenvironment
Source: Br J Cancer. 2022 May 20;127(4):624–36. doi: 10.1038/s41416-022-01853-z (PMC9381763; doi:10.1038/s41416-022-01853-z)
Supplement: Supplementary file 1 — Supplemental Figures [file 41416_2022_1853_MOESM1_ESM.pptx]

## Slide 1
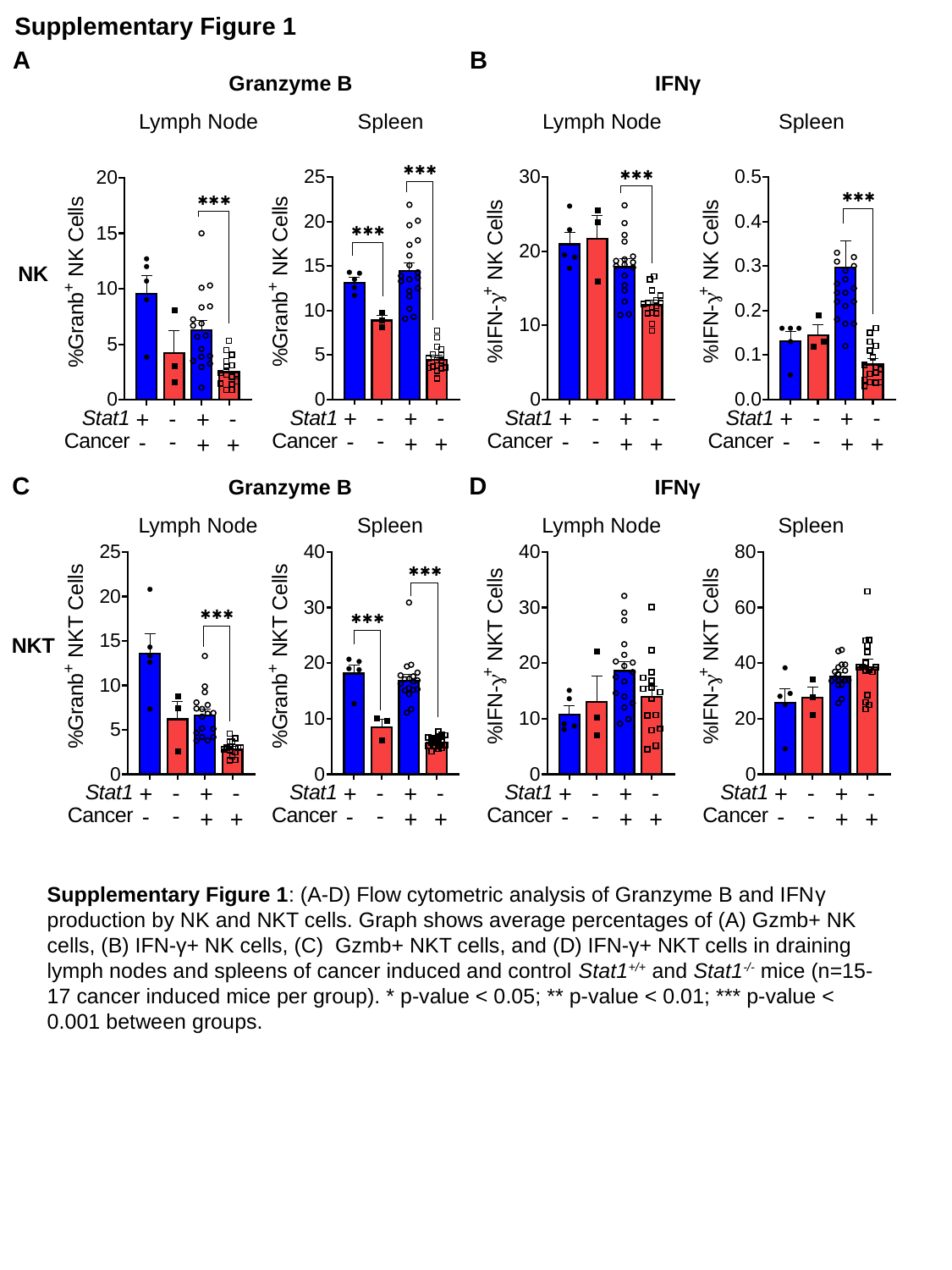

Supplementary Figure 1
A
B
Granzyme B
IFNγ
Lymph Node
Spleen
Lymph Node
Spleen
NK
C
D
Granzyme B
IFNγ
Lymph Node
Spleen
Lymph Node
Spleen
NKT
Supplementary Figure 1: (A-D) Flow cytometric analysis of Granzyme B and IFNγ production by NK and NKT cells. Graph shows average percentages of (A) Gzmb+ NK cells, (B) IFN-γ+ NK cells, (C) Gzmb+ NKT cells, and (D) IFN-γ+ NKT cells in draining lymph nodes and spleens of cancer induced and control Stat1+/+ and Stat1-/- mice (n=15-17 cancer induced mice per group). * p-value < 0.05; ** p-value < 0.01; *** p-value < 0.001 between groups.

## Slide 2
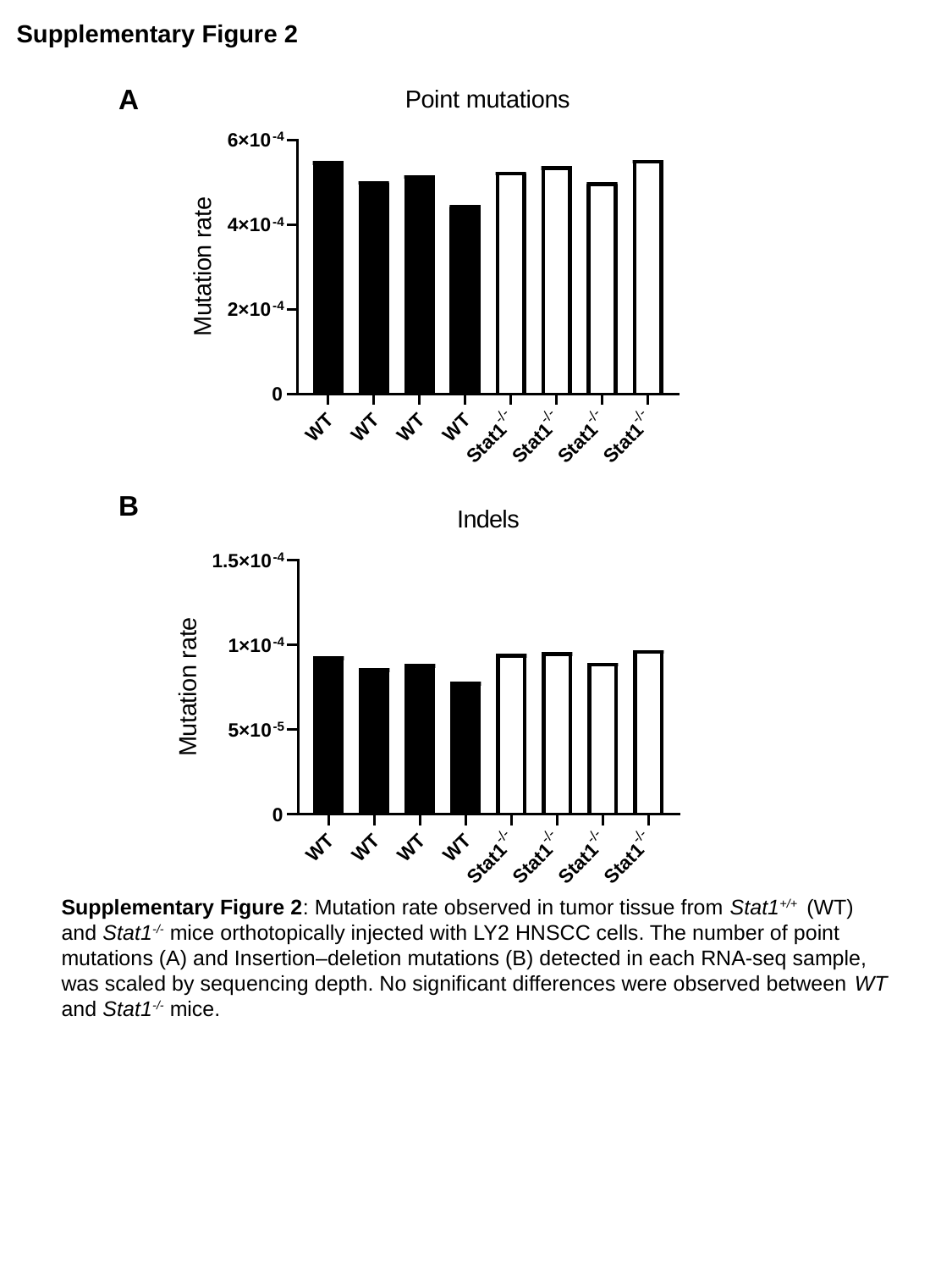

Supplementary Figure 2
A
B
Supplementary Figure 2: Mutation rate observed in tumor tissue from Stat1+/+ (WT) and Stat1-/- mice orthotopically injected with LY2 HNSCC cells. The number of point mutations (A) and Insertion–deletion mutations (B) detected in each RNA-seq sample, was scaled by sequencing depth. No significant differences were observed between WT and Stat1-/- mice.

## Slide 3
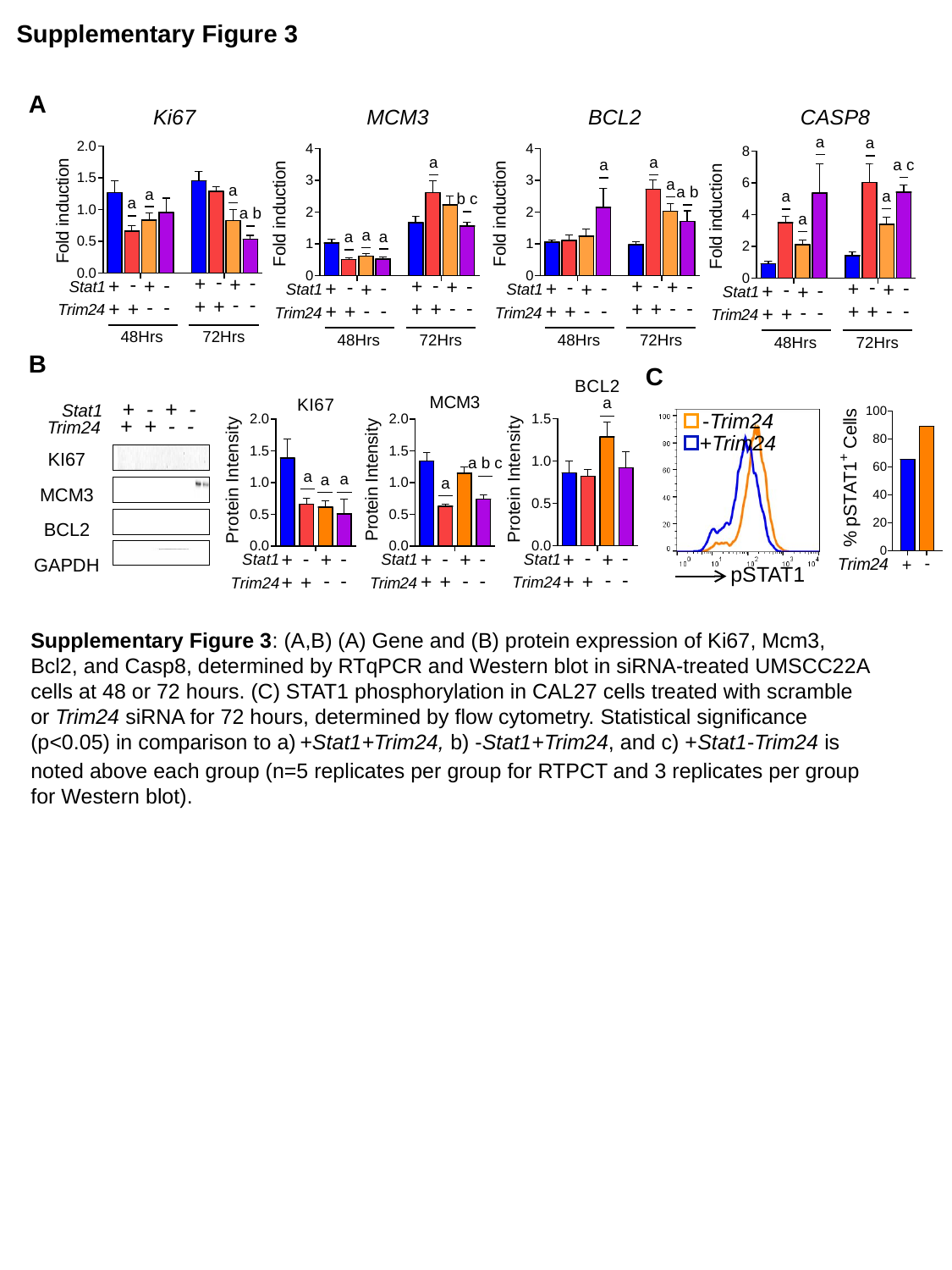

Supplementary Figure 3
A
Ki67 MCM3 BCL2 CASP8
B
C
-Trim24
+Trim24
pSTAT1
 Stat1 + - + -
Trim24 + + - -
KI67
MCM3
BCL2
GAPDH
Supplementary Figure 3: (A,B) (A) Gene and (B) protein expression of Ki67, Mcm3, Bcl2, and Casp8, determined by RTqPCR and Western blot in siRNA-treated UMSCC22A cells at 48 or 72 hours. (C) STAT1 phosphorylation in CAL27 cells treated with scramble or Trim24 siRNA for 72 hours, determined by flow cytometry. Statistical significance (p<0.05) in comparison to a) +Stat1+Trim24, b) -Stat1+Trim24, and c) +Stat1-Trim24 is noted above each group (n=5 replicates per group for RTPCT and 3 replicates per group for Western blot).
